# Measurement of Pesticide Residues from Chemical Control of the Invasive *Spodoptera frugiperda* (Lepidoptera: Noctuidae) in a Maize Experimental Field in Mokwa, Nigeria

**DOI:** 10.3390/ijerph15050849

**Published:** 2018-04-25

**Authors:** Abou Togola, Silvestro Meseka, Abebe Menkir, Baffour Badu-Apraku, Ousmane Boukar, Manuele Tamò, Rousseau Djouaka

**Affiliations:** 1 International Institute of Tropical Agriculture (IITA), PMB 3112, Sabo Bakin Zuwo Road, 700223 Kano, Nigeria; o.boukar@cgiar.org; 2 International Institute of Tropical Agriculture, IITA-HQ Ibadan, PMB 5320, Oyo Road, Ibadan 200284, Nigeria; s.meseka@cgiar.org (S.M.); a.menkir@cgiar.org (A.M.); b.badu-apraku@cgiar.org (B.B.-A.); 3 International Institute of Tropical Agriculture, IITA Cotonou Station, 08 BP 0932, Cotonou, Benin; m.tamo@cgiar.org (M.T.); r.djouaka@cgiar.org (R.D.)

**Keywords:** maize, HPLC, chemical residues, soil contaminant, persistence, fall armyworm

## Abstract

The management of the fall armyworm *Spodoptera frugiperda* in maize field necessitates the use a big quantities of insecticides and sometimes the use of multiple types and formulations of chemicals. The use of insecticides in crops is associated with environmental risks and health hazards to both producers and consumers. This study was designed to evaluate the residue of 11 insecticides that were used to control high population of the fall armyworm in maize field in Mokwa, Nigeria. Maize and soil samples were collected from an experimental field to investigate the residue level using high performance liquid chromatography (HPLC, Agilent Technologies, Santa Clara, CA, USA) analysis techniques. Results revealed the presence of five insecticide compounds (Cypermethrin, Deltamethrin, Lambda-Cyhalothrin, Permethrin, and Chorpyrifos) in soil samples with possible adverse effects on soil born organisms and other non-targeted species. In contrast, no residue was found in maize stems and seeds. From these results, we conclude that the treated maize remains safe for consumption and the producers may not get any serious risk of contamination from the chemical control of the fall armyworm.

## 1. Introduction

Originated from the Western hemisphere from United States, the fall armyworm (FAW) *Spodoptera frugiperda* (Lepidoptera: Noctuidae) is an invasive pest in Africa continent where it was first reported in 2016 [1]. It is now well distributed in many West and Central African countries [1] with regular seasonal outbreaks. According to [2], the fall armyworm was initially detected in Benin, Nigeria, Sao Tome, and Principe, Togo, and the pest has since spread to at least 20 other countries in sub-Saharan Africa. This pest attacks a wide variety of crops, but gramineous plants are most preferred [3], especially maize. During plant vegetative growth stage, larvae primarily feeds on leaves reducing photosynthetic area. Crop seedling are most affected resulting in reduced plant stands and significant yield loss [3]. Staple crops, like maize, sorghum, rice, and sugarcane are severely damaged, but the fall armyworm can ravage more than 80 other plant species with economic losses being estimated at about $13 billion in Africa [2]. Emerging threats from the FAW has severe consequences to smallholder farmers in terms of reduction of income as result of crop yield loss or even total crop failure. The pest is very difficult to control and chemical measure remains the main option in use. Unfortunately, to get good protection of the host crops, farmers need to apply big amount of pesticide as the larvae feed deep in the whorl of maize plant [4] or they need to apply multiple combinations of chemicals. This results in the accumulation of pesticides residues in the environment where they are applied with potential effects on humans, non-target organisms, and biodiversity. Reference [5] postulate that most of the pesticides that are applied in agriculture may distribute into soil sediments, water, and air, and become toxic to organisms that are inhabiting these compartments. Indeed, contamination by pesticide residues constitute a major public issue. The Food and Agriculture Organization of the United Nations (FAO) and the World Health Organization (WHO) have taken action to set permissible residue limits for pesticides and their derivatives. Most of the pesticide manufacturers are subjected to respect chemical production standards. Despite these measures, pesticides residues are found in soil, foods, and other goods due to the misuse, abuse, or overuse making them stored in soil. This study investigates the effects of insecticides in soil, maize stems, and seeds sequel to the use of multiple chemicals for controlling *S. frugiperda.* The study evaluates the residue level in comparison to the Maximum Residue Limit (MRL), which is defined by the International pesticide regulation as authorized standards. This information will be useful for a better use of chemicals against *S. frugperda* that constitutes an emerging threat to maize production in Africa. 

## 2. .Materials and Methods

### 2.1. Maize Plots

The samples for this study were collected from a maize trial planted to eight varieties comprising of six hybrids and two local checks arranged in a randomized complete block design with four replications. The six hybrids (Entry 1, 2, 3, 4, 5, and 6) were developed at IITA and two local checks (Entry 7 and 8) were local varieties that were commonly grown by farmers at Mokwa. The trial was planted during the main rainy season at Mokwa (9°18′ N, 5°4′ E, altitude 457 m) on 8 July 2016. Each entry was planted on a 5 m long and two-row plot with 0.75 m spacing between rows and 0.5 m within rows. The samples were collected only from replication two of the randomized plots to minimize the large size of samples in the whole experiment.

The rainfall pattern at Mokwa is unimodal, it starts in June and ends in October (five months), with dry spell break of two weeks in mid-August (locally known as August break). Though there are sporadic rains in the months of March, April, and May, but the intensity and duration are not good enough to allow for growth and production of maize crop. In 2016, the annual rainfall was 1109 mm, with the maximum averaged daily temperature of 34 °C and minimum of 22 °C. This amount of rainfall and range of daily temperatures helped the growth of maize in Mokwa. 

### 2.2. Chemical Application

Multiple chemicals were consecutively applied to control the FAW. The chemical application protocol was similar to that commonly used by farmers. No history of previous chemical application in the experimental field was recorded during the past 10 years. The list of chemicals and periods of their application are shown in Table 1.

The sprayer used for this experiment was a Knapsack CP3, 20 L. The nozzle that was used was the insecticides type (hollow cone Yellow). The spray width was 2.30 inches with a maximum spraying pressure of 300,000 Pascal.

Prior to the insecticide application, the sprayer was calibrated while considering the time that was taken to walk a fixed distance, the flow rate and the spray width. The calculated volume was 220 L of mixed solution per ha in which the dosage rate of each chemical was defined as recommended in the label.

### 2.3. Sampling Methods:

Maize grains and stems and also soils samples were collected from eight randomly selected plots in the experimental field. 

The sampling method consisted of collecting:

- 10 g of grains from five different randomly selected plants of each entry in a plot,
- 10 cm piece of stem from five different randomly selected plants of each entry in a plot, and
- five soil samples of 10 g at 20 cm deep in different randomly selected areas in each plot.

The samples were kept in separate plastic bags, labeled, and sent to the AgroEcoHealth Platform laboratory at the International Institute of Tropical Agriculture Cotonou station (Cotonou, Benin) for pesticide residues analysis.

### 2.4. Protocol of Pesticide Residues Analysis

The investigation was essentially about soil and maize seeds samples, but some samples of commercial products of the used pesticides were also submitted to the analysis to compare the chromatograms of the active ingredients of the residues with the standards. The samples were analyzed to confirm the Maximum Residue Limits (MRL), the Acceptable Daily Intake (ADI) and the Acute Reference Dose (ARfD).

The method of analysis was the following:

- Pesticide residues in soil samples were analyzed using Reverse Phase HPLC (Agilent Technologies, Santa Clara, CA, USA) with C18, 5 µm 120 Â, 4.6 × 250 mm column as stationary phase and methanol/H_2_O (90:10) as mobile phase at a flow rate of 1 mL/min for 30 min.
- Five (5) insecticides standards, which are part of the active ingredients in the commercial products, were used as reference and for quantifications: Cypermethrin, Permethrin, Deltamenthrin, Lambdacyhalothrin, and Chorpyrifos.
- Duplicates of the soil samples were examined. Extraction was done with 1 mL acetonitrile and 1 g soil sample using Microwave Extraction method. Each replicate was divided into 2, and one was spiked with prepared cocktail of the standards. Thus, the total run per sample on the HPLC was 4.
- The duplicates and the corresponding spiked samples were overlaid for the identification of possible insecticide residues.
- Duplicates of the maize samples were examined. 5 g of maize first was grinded to powdered form. Slurry was made from the powder by blending in 10 mL of ddH_2_O water. 10 mL acetonitrile was added and then shaken at 8 rpm for 30 min to allow for the repartitioning of the residues of the insecticides into acetonitrile. A modified QuECHERS method was used to separate the water/acetonitrile phases. 8 mL of the acetonitrile was then concentrated to 1 mL and use for the HPLC analysis.
- Area of peaks were used for the standard curves of the pyrethroid insecticides, while the height of peaks used for the Chlorpyrifos-Ethyl.

#### 2.4.1. The LOD, LOQ Recovery, Precision and Accuracy

Limit of detection/limit of quantification (LOD/LOQ) was previously done with lambda Cypermtherin using the linear regression method [6]. The standard solutions were also spiked into the test samples to estimate the percentage recovery and to identify the respective insecticides. The standard curve calibration was performed with six points and a retention time (RT) of 0.999. 

#### 2.4.2. Chromatography and HPLC Analysis

The Chromatogram peaks of the active ingredients were identified by spiking the experimental samples. Samples were ran on HPLC without spiking, and the chromatogram overlaid on that of a standard. The active ingredient were identified within limit of detection (LOD), but below the limit of quantification (LOQ) of the HPLC. We ran some samples of the commercial products on HPLC, but other non-insecticidal ingredients were distorting the chromatographic peaks. We also spiked the commercial products with purified insecticide samples to track their presence and to quantity them using HPLC. The active ingredients in the commercial products are presented in Table 1.

#### 2.4.3. Quality Assurance/Quality Control (QA/QC) Information

The samples were sent for analysis properly protected from ultraviolent radiation from sun to prevent the degradation of the insecticides and kept in the fridge prior to analysis carried out within a week from collection. Eleven commercial products were used and the active ingredients were similar in most products or combined. The five insecticides that were used for the calibration preparation (Cypermethrin, Permethrin, Deltamenthrin, Lambdacyhalothrin, and Chorpyrifos) are good representatives of the active ingredients in the 11 commercial products.

## 3. Results 

### 3.1. Detection of Chemical Compound in Soil Samples

The results from the HPLC analysis of soil samples revealed an evident presence of pyrethroid class compounds, such as Cypermethrin, Lambda-Cyhalothrin, Deltamethrin, and Permethrin. The most common ingredients that were found in soil samples were Cypermethrin, Lambda-Cyhalothrin, and Deltamethrin. These compounds were detected in Plot 15/ENTRY 1, Plot 10/ENTRY 4, Plot 9/ENTRY 6, Plot 11/ENTRY 7, and Plot 13/ENTRY 8 (Table 2).

The retention times (RT) of the detected chemical compounds were 7.14 min for Lambda-Cyhalothrin, 8.09 and 8.66 min for Cypermethrin (Cis) and (Trans), 8.51 for Deltamethrin, and 11.63 and 13.93 min for Permethrin (Trans) and (Cis). The RT values of some compounds were very close, particularly that of Lamda Cyhalothrin, as well as that of Permethrin and Cypermethin. In contrast, the RT values of some isomers of the same compounds were significantly different, as shown by Permethrin Trans and Cis (Table 3).

#### 3.1.1. Detection of Cypermethrin

**Figure 1:**
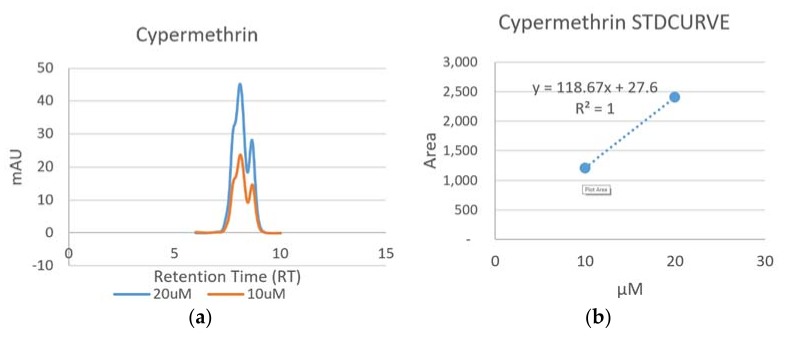
Chromatogram (**a**) and standard curve (**b**) of Cypermethrin.

The chromatogram and the standard curve of Cypermethrin are presented in Figure 1. Two isomers (Cis and Trans) of this compound with retention times of 8.09 and 8.66 min, respectively, were found (Figure 1a). The regression equation was y = 118.67x + 27.6 with a correlation coefficient of 1 (Figure 1b).

#### 3.1.2. Detection of Lamda-Cyhalothrin 

**Figure 2:**
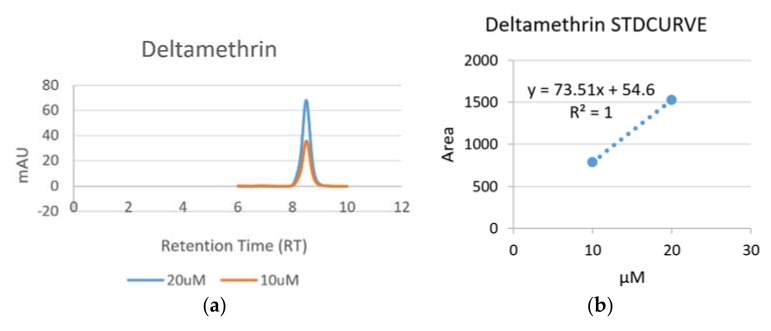
Chromatogram (**a**) and standard curve (**b**) of Deltamethrin.

The chromatogram and the standard curve of Lamda-Cyhalothrin were generated as shown below (Figure 2). The retention time of this compound for the HPLC analysis was 7.136 min (Figure 2a). The regression equation was y = 115.04x + 47.4, with a correlation coefficient of 1 (Figure 2b).

#### 3.1.3. Detection of Deltamethrin

**Figure 3:**
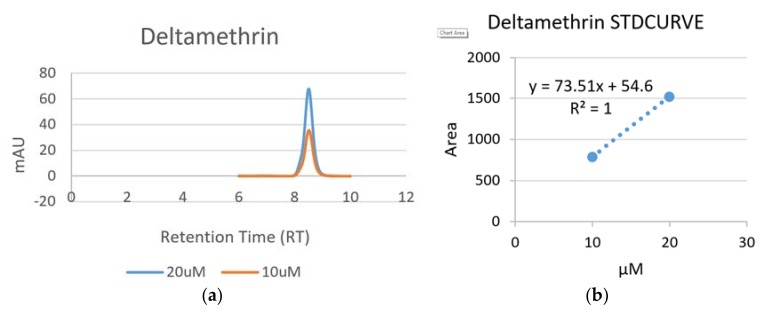
Chromatogram (**a**) and standard curve (**b**) of Deltamethrin.

The chromatogram and standard curve of Deltamethrin are presented below (Figure 3). The retention times of Deltamethrin was 8.151 min (Figure 3a). The regression equation was y = 73.51x + 54.6, with a correlation coefficient of 1 (Figure 3b).

#### 3.1.4. Detection of Permethrin

**Figure 4:**
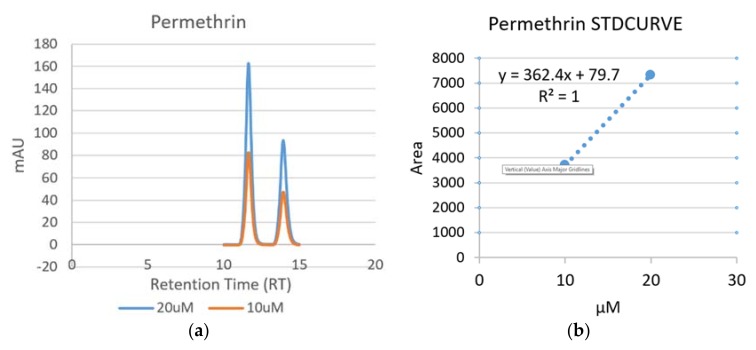
Chromatogram (**a**) and standard curve (**b**) of Permethrin.

The chromatogram and standard curve of Permethrin are presented in Figure 4. Two isomers (Trans and Cis) of this compound were found with retention times of 11.63 and 13.925 min, respectively (Figure 4a). Permethrin regression equation was y = 362.4x + 79.7, with a correlation coefficient of 1 (Figure 4b). 

**Figure 5:**
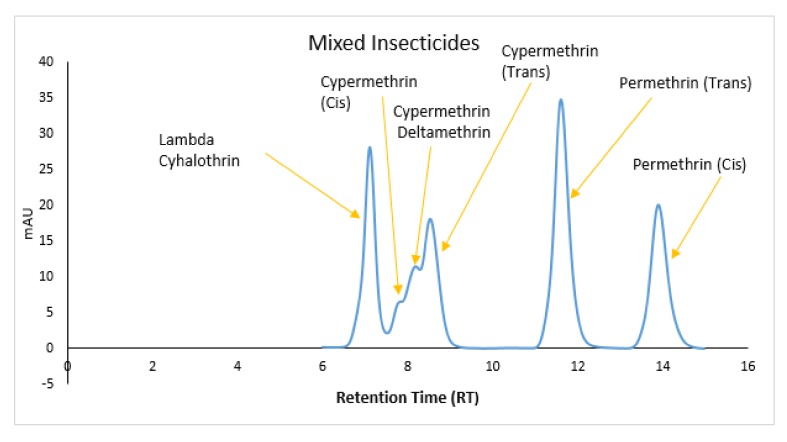
Retention times peaks of the active ingredients detected in soil samples.

The chromatogram generated from the mixed insecticides showed close RT values of Lambda-Cyhalothrin and those of Cypermethrin Cis and Deltamethrin. No peak of both isomers of permethrin was seen, although some peaks around the RTs were detected (Figure 5).

**Figure 6:**
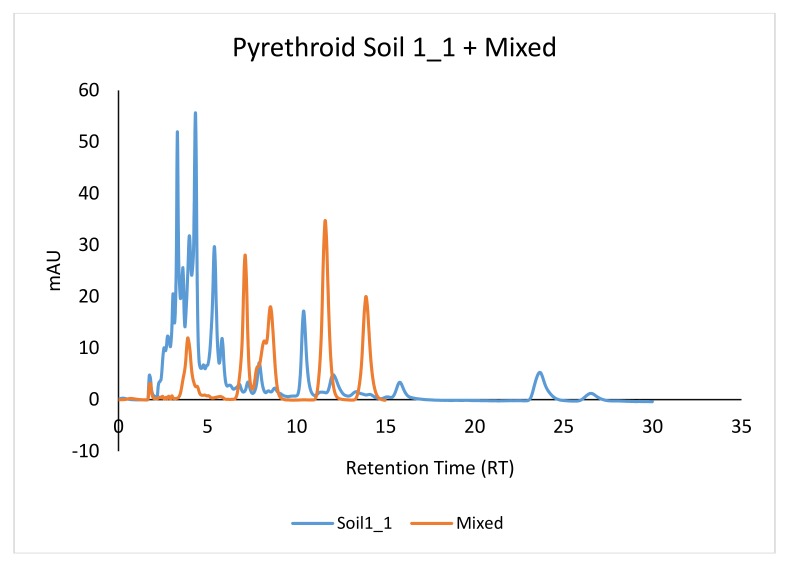
Retention times of the active ingredients detected in soil samples.

Other noticeable peaks were seen under RTs 15, 23, and 28. These peaks correspond not only to the β Cyfluthrin from pyrethroid class, but also to other unknown agrochemicals that were detected at 226 nm (Figure 6). Some of these detected insecticides were not among the pesticides that were used in this study, but may be from previous treatments.

We noted that the HPLC analysis did not reveal any presence of Dichlorvos, Profenofos/Citric Acid, Dichlorvos, Dimethoate, β Cyfluthrin, and Pirimiphos-methyl in the soil sample, despite these compounds being involved in the control of the FAW.

### 3.2. Detection of Chemical Compound in Maize Stem and Seeds Samples

The HPLC analysis did not show any of the compounds of the used chemical in maize stems and seeds.

## 4. Discussion

This study has showed the evidence of the presence of Cypermethrin, permethrinand Deltamethrin residues in the soil of the treated maize fields. These chemicals belong to the synthetic pyrethroid class. Indeed, pyrethroid chemicals represent the most widely used insecticides by farmers for protecting field crops against various insect species in Nigeria. Among this class of insecticides, Cypermethrin, λ-cyhalothrin, and Deltamethrin are the main active ingredients that are used in large scale by local farmers for controlling the FAW. Very often they are used without much respect regarding the dose or the remanence periods. Other compounds, such as Deltamethrin, Permethrin, and Chlorpyrifos-ethyl were also found in the soil. The extent and duration of the pesticides residue following spray drift contamination of soil and plants depend on the physio-chemical and insecticidal characteristics of the compound. 

Cypermethrin is known to be a synthetic pyrethroid that is used in large-scale as agricultural insecticide. It degrades easily in soil and plants. We found it in the soil samples of maize field following the chemical treatment against the FAW. This may have resulted from the used of inadequate doses for controlling the FAW. Although any noticeable risk is expected in humans, it is considered as a broad-spectrum insecticide that is capable of killing both beneficial insects and targeted insects [7]. It was reported to be highly toxic for some non-targeted species, such as bees, aquatic insects, and fish [8], but when used according to rights prescriptions and dosage, it poses little risk [5]. Therefore, it will be advisable to use it adequately when managing the FAW to avoid not only the increase of the toxic effect to non-targeted organisms, but also the risk of resistance in the pest. According to [9], resistance to cypermethrin can develop quickly in insects exposed frequently and can render it ineffective. Early studies reported that *S. frugperda* has developed resistance to the majority of insecticides classes [10,11,12,13].

Deltamethrin is a pyrethroid ester insecticide that has many uses, ranging from agricultural uses to home pest control. It is generally considered to be safe to use around humans, although its neurotoxic effects were reported in [4]. In South Africa, residues of deltamethrin were found in an area where it was used for malaria control, as well as in small-scale agriculture [14]. According to [15] it is highly toxic to aquatic life, particularly fish, and therefore it must be used with extreme caution around water.

Permethrin is used in agriculture as contact insecticide to protect crops. It is mainly used on cotton, wheat, maize, and alfalfa crops. It was reported to be a broad-spectrum chemical with lethal effect to intended pests and it also to beneficial insects, including honey bees and aquatic life [16].

Lambda-Cyhalothrin is a mixture of isomers of cyhalothrin, a synthetic organic insecticide [17]. It is used as a broad-spectrum pyrethroid insecticide to control a wide range of insect pests in a variety of crops [18]. Lambda-Cyhalothrin has low potential to contaminate ground water due to its low water solubility and high potential to bind to soil [19]. Sunlight can accelerate its degradation in both water and soil, and its half-life on plant surfaces is approximatively five days [20]. Hence, the residue that was detected in this study might be of moderate or slight risk for the biodiversity of the ecosystems. However, due to the high toxicity to aquatic organisms, necessary precaution should be taken when using lambda cyhalothrin near a water source.

Chlorpyrifos (CPS) is an organophosphate pesticide that is used to kill a number of pests, including insects and worms [21]. It is considered as moderately hazardous to human [22] because of its persistent health hazards, acute poisoning, or its developmental effects in fetuses or children after long-term exposure, even to very small doses [23]. Aquatic insects and animals appear to absorb chlorpyrifos directly from water or through sediment exposure [24]. High health risk is primarily due to acute exposure to chlorpyrifos. A study conducted by [25] revealed the chronic effect from low-level exposure through residue in pollen and components of bee hives, such as honey. 

In sum, we noted that the detected residues were found only in soil samples, whereas the maize stems and grains were free of residue. This is because most of the insecticides that were used were essentially pyrethroids insecticides (except the chlorpyrifos) without any persistence.

Pyrethroid pesticides have short lifespan and relative low toxicity. They have a quick degradability of compounds and a low risk on the environment [26]. However, the misuse, abuse, or overuse of these pesticides can make them stored in soil and make them toxic to soil born-organisms, even at low levels. Moreover, these products in soil can contaminate irrigated or streaming rainfall water [27,28] from treated fields. In this study, the number of pesticides that were used was unrealistically high because the first chemicals applied were not effective in controlling the FAW and the population of the pest was getting higher. Thus, the need for additional stronger chemicals was required. The most important lesson to learn from this study is the risk of soil contamination that results from the chemical control of the FAW. According to the sample types where the residues are stored and according to the specificities of the residue compounds, there is very little risk for human beings, but the hazards to soil born organisms and others non-targeted species remain high. Therefore, to minimize the issue of residue and to avoid the increase of pesticide-resistance in *S. frugiperda*, we recommend the choice of appropriate selective insecticides and their proper use following reasoned directions. Also, we recommend the integration of other options, such as biological control, use of bio-pesticides, and host plant resistance for the management of FAW. Above all, special attention should be paid on the use of prophylactic measures of which the early monitoring appears to be very informative for knowing the initial infestation of the pest when the density is still low.

## 5. Conclusions

This study has provided evidence of the accumulation of some pesticides compounds residues in soil samples from maize field sequel to the chemical control of the fall armyworm *Spodoptera frugiperda*. It has also elucidated the absence of residue in the crop samples. The incriminated causes of the residue accumulation in soils were the misuse, abuse, or overuse of the pesticides. The main risks that were raised from the study were the possible adverse effects of the residues on soil born organisms, bees, and aquatic species at one hand, and the increase of pesticide resistance to the fall armyworm on the other hand. To minimize these risks, the study has proposed an integrated management approach, including early monitoring, biological control, use of bio-pesticides, host plant resistance, and the use of appropriate chemical at reasonable doses for controlling the invasive fall armyworm.

## Figures and Tables

**Table 1:** List of chemicals, their active ingredients, and quantity applied in field.

Date of Application	Chemical	Active Ingredients	Quantity	Plots
27–31/7/2016	PYRINEX and and BEST ACTION	Chlorpyrifos/Xylene	15 mL each	Experiment plots
1–25/8/2016	CRUSH and MAGIC FORCE	Cypermethrin/Dimethoate	20 mL and 30 mL	Experiment plots
26/8/–8/9/2016	DD FORCE and SHARP SHORTER	β Cyfluthrin	40 mL and 30 mL	Experiment plots
9–12/9/2016	ATTACK and COURAGE	Lambda-Cyhalothrin/Dimethoate	50 mL and 30 mL	Experiment plots
13–15/9/2016	GOODBYE and SHARP SHOOTER	Dichlorvos	30 mL and 70 mL	Experiment plots
16–19/9/2016	GOODBYE and PYRINEX	Citric Acid	30 mL and 70 mL	Experiment plots
20–22/9/2016	V.I.P and SHARP SHOOTER	Permethrin/Pirimiphos-methyl	30 mL and 70 mL	Experiment plots
23/9–9/10/2016	DD FORCE and IRON FORCE	Imidacloprid	30 mL and 70 mL	Experiment plots
10–28/10/2016	IRON FORCE	Pyrethrum	100 mL	Seed multiplication
22–24/11/2016	PYRINEX	Profenofos/Cypermethrin	100 mL	Seed multiplication

**Table 2:** The active ingredients that were detected in soils samples.

SN	Sample Name	No of Replicates Tested	Active Ingredients Detected
1	Plot 15/ENTRY 1	2	Lambda/Cyperm
2	Plot 14/ENTRY 2	2	Cyperm/Delta
3	Plot 12/ENTRY 3	2	Perm
4	Plot 10/ENTRY 4	2	Lambda/Cyperm
5	Plot 16/ENTRY 5	2	Cyperm/Delta
6	Plot 9/ENTRY 6	2	Lambda/Cyperm/Delta
7	Plot 11/ENTRY 7	2	Lambda/Cyperm/Delta
8	Plot 13/ENTRY 8	2	Lambda/Cyperm/Delta

Cyperm = Cypermethrin; Perm = Permethrin; Lambda = Lambda − cyhalothrin; Delta = Deltamethrin.

**Table 3:** Retention time of the main pesticide ingredients detected in soil.

Sample Name	Retention Time
Lambda-Cyhalothrin	7.14
Cypermethrin (Cis)	8.09
Cypermethrin (Trans)	8.66
Deltamethrin	8.51
Permethrin (Trans)	11.63
Permethrin (Cis)	13.93
